# Integrated analysis of miRNA, gene, and pathway regulatory networks in hepatic cancer stem cells

**DOI:** 10.1186/s12967-015-0609-7

**Published:** 2015-08-11

**Authors:** Min Ding, Jiang Li, Yong Yu, Hui Liu, Zi Yan, Jinghan Wang, Qijun Qian

**Affiliations:** Department of Viral and Gene Therapy, Eastern Hepatobiliary Surgery Hospital, The Second Military Medical University, Shanghai, 200438 People’s Republic of China; The First Department of Biliary Surgery, Eastern Hepatobiliary Surgical Hospital, The Second Military Medical University, Shanghai, 200438 People’s Republic of China

**Keywords:** Hepatic cancer stem cells, Hepatic cancer cells, miRNA, mRNA, Pathway analysis, Regulatory network

## Abstract

**Background:**

Hepatocellular carcinoma (HCC) is one of the most common malignancies worldwide. HCC has a poor prognosis associated with tumor recurrence and drug resistance, which has been attributed to the existence of hepatic cancer stem cells (HCSCs). However, the characteristics and regulatory mechanisms of HCSCs remain unclear. We therefore established a novel system to enrich HCSCs and we demonstrate that these HCSCs exhibit cancer stem cell properties.

**Methods:**

We used miRNA and mRNA high-throughput sequencing data sets to determine molecular signatures and regulatory mechanisms in HCSCs. Paired miRNA and gene deep sequencing data in HCSCs versus HCC cells were used to identify candidate biomarkers of HCSCs. Using network analysis, we studied the relationship between miRNA and gene biomarkers, and KEGG pathway enrichment analysis was performed to study the function of candidate biomarkers.

**Results:**

We identified 9 up- and 9 down-regulated miRNAs and 115 up- and 402 down-regulated genes in HCSCs compared with HCC cells. A miRNA-gene network was constructed using 651 miRNA–gene interactions (between 7 up-regulated miRNAs and 274 down-regulated genes), and 103 miRNA–gene interactions (between 9 down-regulated miRNAs and 62 up-regulated genes). Pathway enrichment analysis identified five tumor invasion- and metastasis-related pathways and MAPK signaling associated with HCSCs. We further discovered two novel pathways that likely play a role in the regulation of HCSCs.

**Conclusions:**

We identified a molecular expression signature and pathway regulatory mechanisms in HCSCs with potential diagnostic and therapeutic value.

**Electronic supplementary material:**

The online version of this article (doi:10.1186/s12967-015-0609-7) contains supplementary material, which is available to authorized users.

## Background

Hepatocellular carcinoma (HCC) is one of the most common malignancies that accounts for 70–85% of liver cancers worldwide [1]. Although significant progress has been made in recent years regarding the treatment options for HCC, poor prognosis remains a problem because of late diagnosis, recurrence, and drug resistance [2]. While surgical intervention–the main treatment option for HCC–is effective in patients diagnosed at an early stage [3], the treatment of advanced liver cancer is more difficult and prognosis remains poor because of drug resistance [4], making recurrence almost inevitable [5]. Cancer stem cells (CSCs), which are critical for the initial growth and maintenance of the tumor, have been identified in liver cancers [6–8]. Recently, CSCs have been associated with tumor recurrence and drug resistance in HCC [9, 10]. CSCs are potential targets for HCC diagnosis and treatment–it is therefore crucial that we study the regulatory mechanisms of CSCs.

Several biological markers of hepatic cancer stem cells (HCSCs), including CD133 [10], CD90 [11], and EpCAM [12] have been identified. However, the characteristics and regulatory mechanisms of HCSCs remain unclear. We therefore established a novel system to enrich HCSCs and previously reported that these cells have CSC characteristics [13].

MicroRNAs (miRNAs), a class of small non-coding RNAs, have been shown to play an important role in a variety of biological processes. Abnormal expression of miRNAs may impact the expression of hundreds of genes. Recently, with the development of microarray and high-throughput sequencing technology, miRNA–mRNA interactions in cancers and other biological processes have been extensively studied [14–16]. However, genome-wide miRNA–mRNA interactions in HCSCs remain largely unknown.

**Fig. 1:**
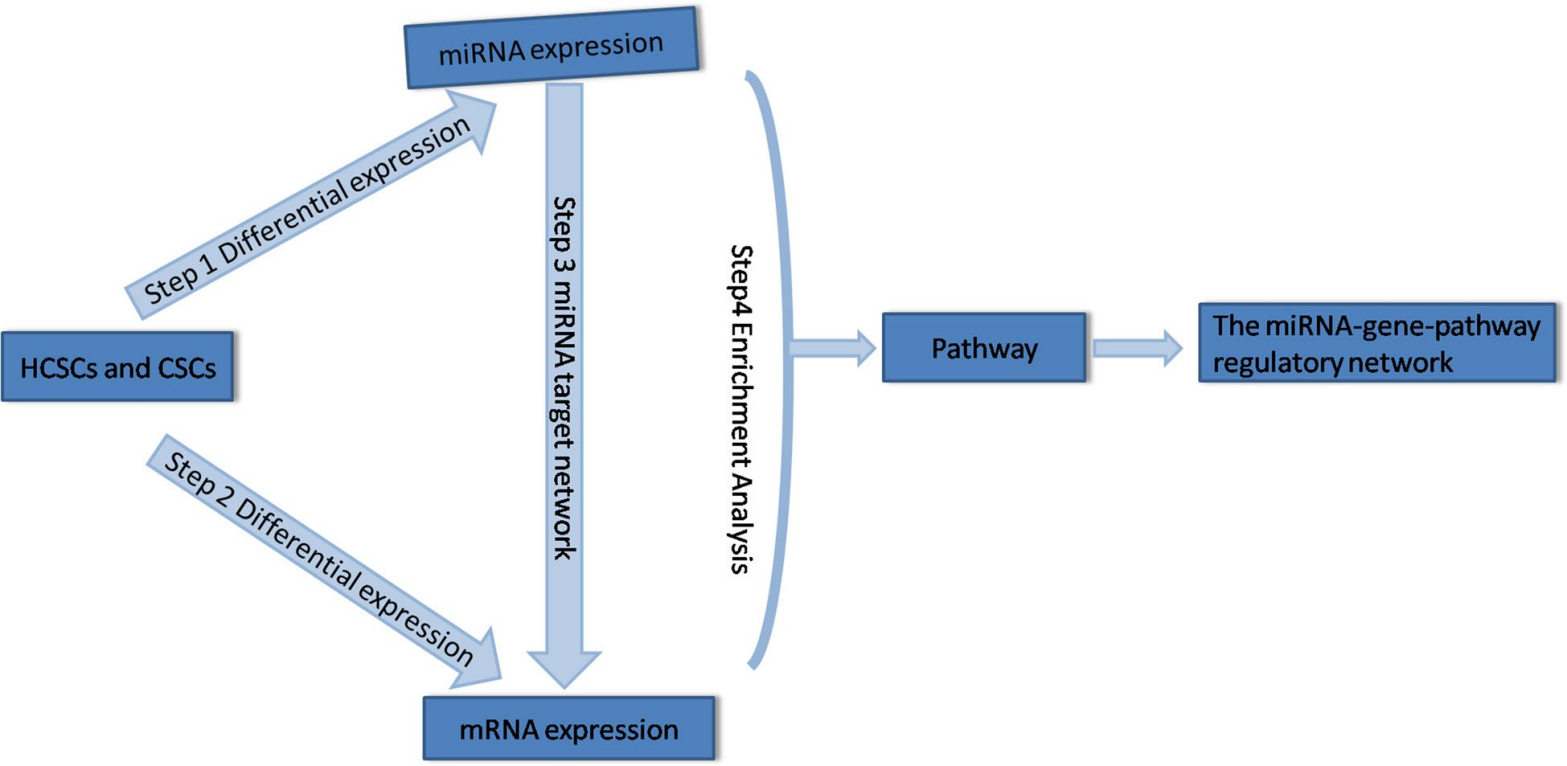
The network analysis pipeline.

In this study, we performed high-throughput sequencing of HCSC small RNA and mRNA, and integrated miRNA and mRNA data to identify biomarkers of HCSCs and to unravel HCSC regulatory networks. Our network analysis approach is summarized in Fig. 1.

## Methods

### Data

We used two human hepatoma cell lines (Hep3B and Huh7) from the American Type Culture Collection (ATCC) to culture stem-like cancer cells. We previously demonstrated that these cells have enhanced stem cell properties, drug resistance, properties of epithelial mesenchymal transition, and enhanced tumor-initiating capabilities [13]. Here, we analyzed the regulatory mechanism of these two types of HSCSc using miRNA and RNA sequencing data. MiRNA and gene expression data of hepatic cancer stem cells and cancer cells were sequenced using the Illumina Genome Analyzer, to a depth of 30-fold coverage. Details of the data sets are shown in Table 1.

**Table 1:** Details of data sets

Data type	Cell line	Total reads	Normalized
miRNA	Hep3B-C	15440438	TPM
	Huh7-C	10714837	
	Hep3B	16782602	
	Huh7	10622276	
RNA	Hep3B-C	5762058	RPKM
	Huh7-C	5886142	
	Hep3B	6116344	
	Huh7	5998659	

Paired miRNA and gene deep sequencing datasets from the same sample of two hepatic cancer stem cells and two hepatic cancer cells were used. Detailed information about these datasets is provided in this table.

Small RNA and mRNA libraries were sequenced on an Illumina Genome Analyzer II (Illumina, San Diego, CA, USA) according to the manufacturer’s instructions. Raw RNAseq reads were filtered for adapters and/or low-quality reads, followed by alignment to the human genome (NCBI Build 36.1) using NextGENe^®^ software (Softgenetics, State College, PA, USA). Reads mapping to individual transcripts were counted digitally, and the expression levels for each gene were normalized using reads per kilobase of exon model per million mapped reads (RPKM) [17]. For small RNA analysis, after filtering contaminant and redundant reads, and reads smaller than 18 nt, clean reads were mapped to the human genome (NCBI Build 36.1) using SOAP [18]. Reads were then mapped to Ribosomal RNA (rRNA), Small cytoplasmic RNA (scRNA), small nuclear RNA (snRNA), small nucleolar RNA (snoRNA), and transfer RNA (tRNA) from GenBank. The remaining reads were searched against miRBase (release 17) [19]. Read counts of the annotated miRNAs were normalized using transcripts per million (TPM) [20].

### Differential expression analysis

Genes and miRNAs differentially expressed between hepatic cancer stem cells and hepatic cancer cells were identified by calculating fold change values and using an established statistical method based on the Poisson distribution [21–23] to calculate p-values. The Benjamini–Hochberg FDR method [24] was used to adjust p-values for multiple testing. MiRNAs and genes with an absolute log2 fold-change (expression of cancer stem cells/expression of cancer cells) ≥1 and an FDR ≤0.01 were considered statistically significant.

### miRNA target prediction

Using the results from differential gene and miRNA expression (cancer stem cells vs. cancer cells), gene–miRNAs interactions were predicted using seven miRNA target computational prediction methods: MicroCosm (Version 5) [25], microT (version 3) [26], miRanda (August 2010 available at http://www.microrna.org/microrna/home.do) [27], miRDB (version 4.0) [28], PicTar (four-way) [29], PITA (version 6) [30], and TargetScan (version 5.2) (with total context score >−0.3) [31]. Except for TargetScan, we used default cut-off values. Interactions that occurred in at least two of these sources were considered for downstream analyses.

### Pathway enrichment analysis

Pathway enrichment analysis was employed to investigate the regulatory mechanisms of significantly differentially expressed miRNAs. KEGG and DAVID Bioinformatics Resources 6.7 databases were used for pathway enrichment analyses. The enriched pathways were defined by their enrichment of significantly differentially expressed miRNA target genes. For DAVID functional annotation, the Fisher’s exact test was used to calculate statistical significance (p values) of enriched annotation terms, where a smaller *p* value implies enrichment, and a p-value ≤0.05 was deemed significant.

## Results

### Identification of candidate biomarkers in hepatic cancer stem cells

Differential expression analysis was used to identify candidate biomarkers in hepatic cancer stem cells. Using deep sequencing, miRNAs and genes differentially expressed between stem cells (Hep3B-C, Huh7-C) and the paired cancer cells from which they were derived (Hep3B, Huh7) were identified. Data normalization and differential expression analysis are described in the “Methods” section.

**Fig. 2:**
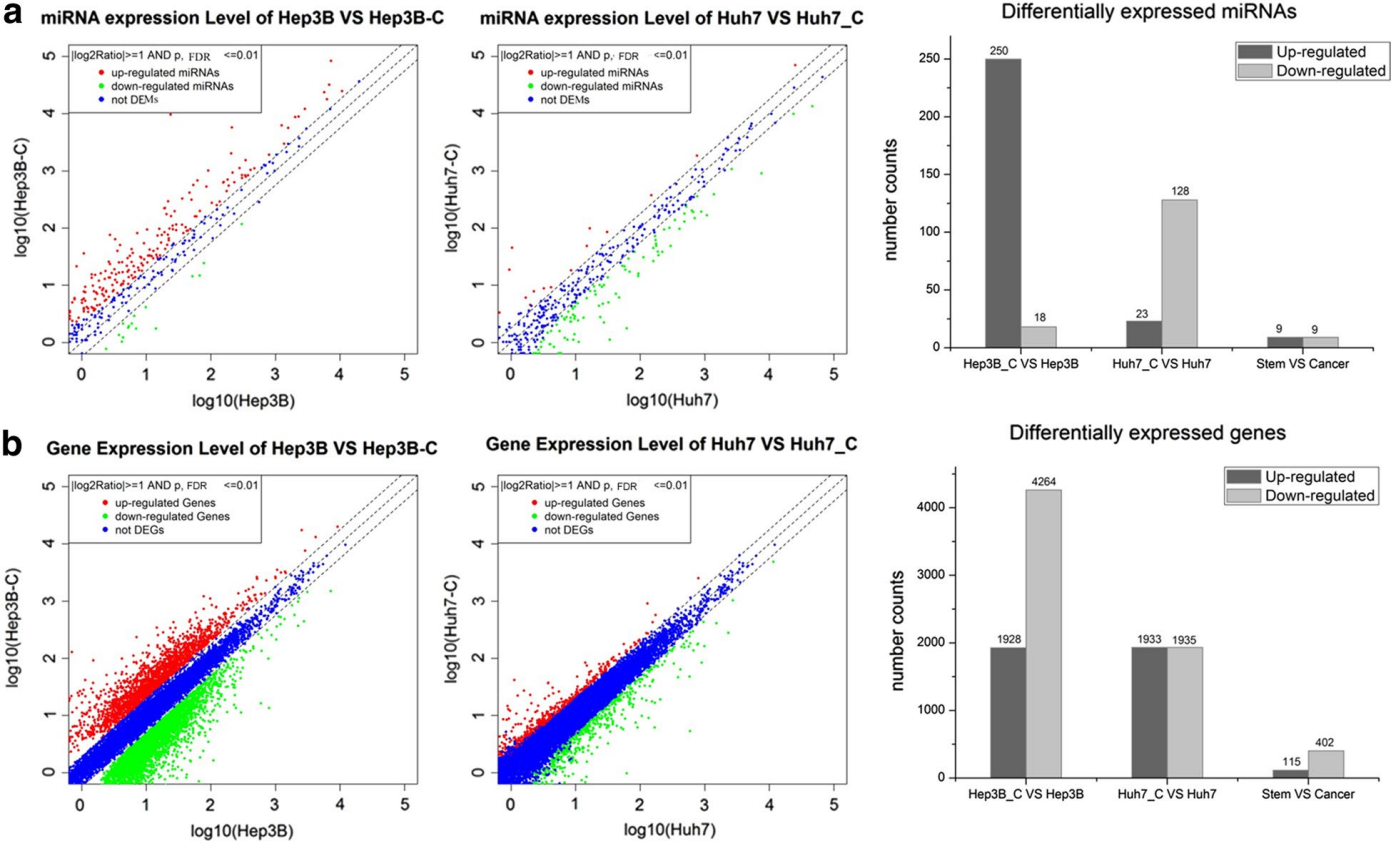
Differentially expressed miRNAs and genes. **a**, **b** illustrate the selection of differentially expressed miRNAs and genes, respectively between stem and cancer cells. Two paired stem- and cancer-cell samples (Hep3B-C, Hep3B) and (Huh7-C, Huh7) were used here; miRNAs and genes consistently up- or down-regulated in both sample pairs were selected.

For miRNAs, 250 (59.7%) up- and 18 (4.3%) down-regulated miRNAs were identified in Hep3B-C cells, while 23 (5.4%) up- and 128 (30.2%) down-regulated miRNAs were identified in Huh7-C cells. Finally, we selected 9 up- and 9 down-regulated miRNAs, that were consistently altered in both stem cell lines (compared with their corresponding cancer cells), as candidate miRNA biomarkers of HCSCs (Fig. 2a). Using the same two paired samples, differential gene expression analysis was performed, which produced 1928 (13.0%) up- and 4264 (28.8%) down-regulated genes in Hep3B-C (vs. Hep3B) cells and 1933 (13.1%) up- and 1935 (13.1%) down-regulated mRNAs in Huh7-C (vs. Huh-7) cells. As shown in Fig. 2b, 115 and 402 genes were consistently up- and down-regulated, respectively in the two stem cell lines; these miRNAs and genes were used for downstream analyses (Additional file 1: Table S1, Additional file 2: Table S2).

### miRNA-gene regulatory network analysis

**Fig. 3:**
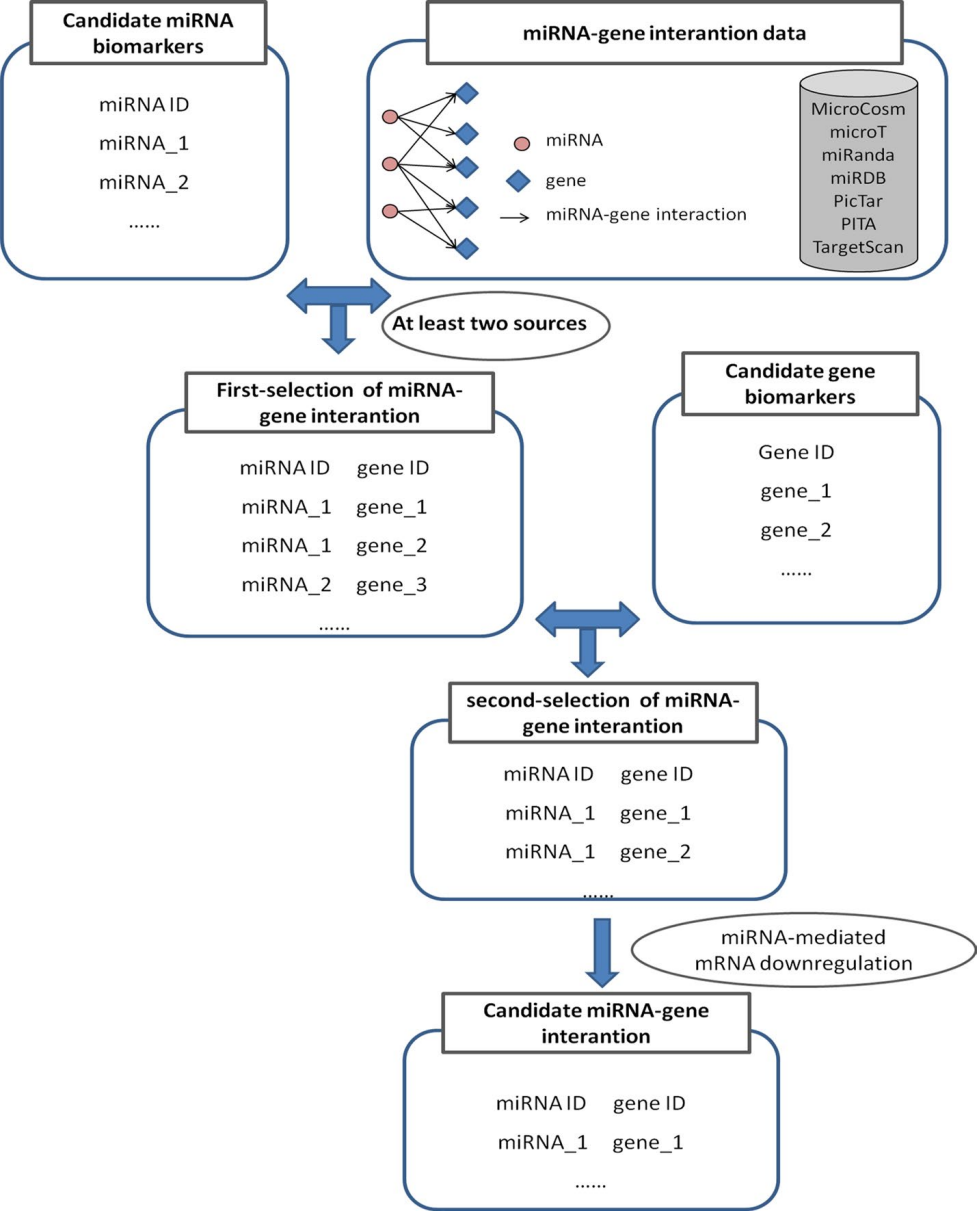
Workflow for the selection of candidate miRNA–gene interactions.

**Fig. 4:**
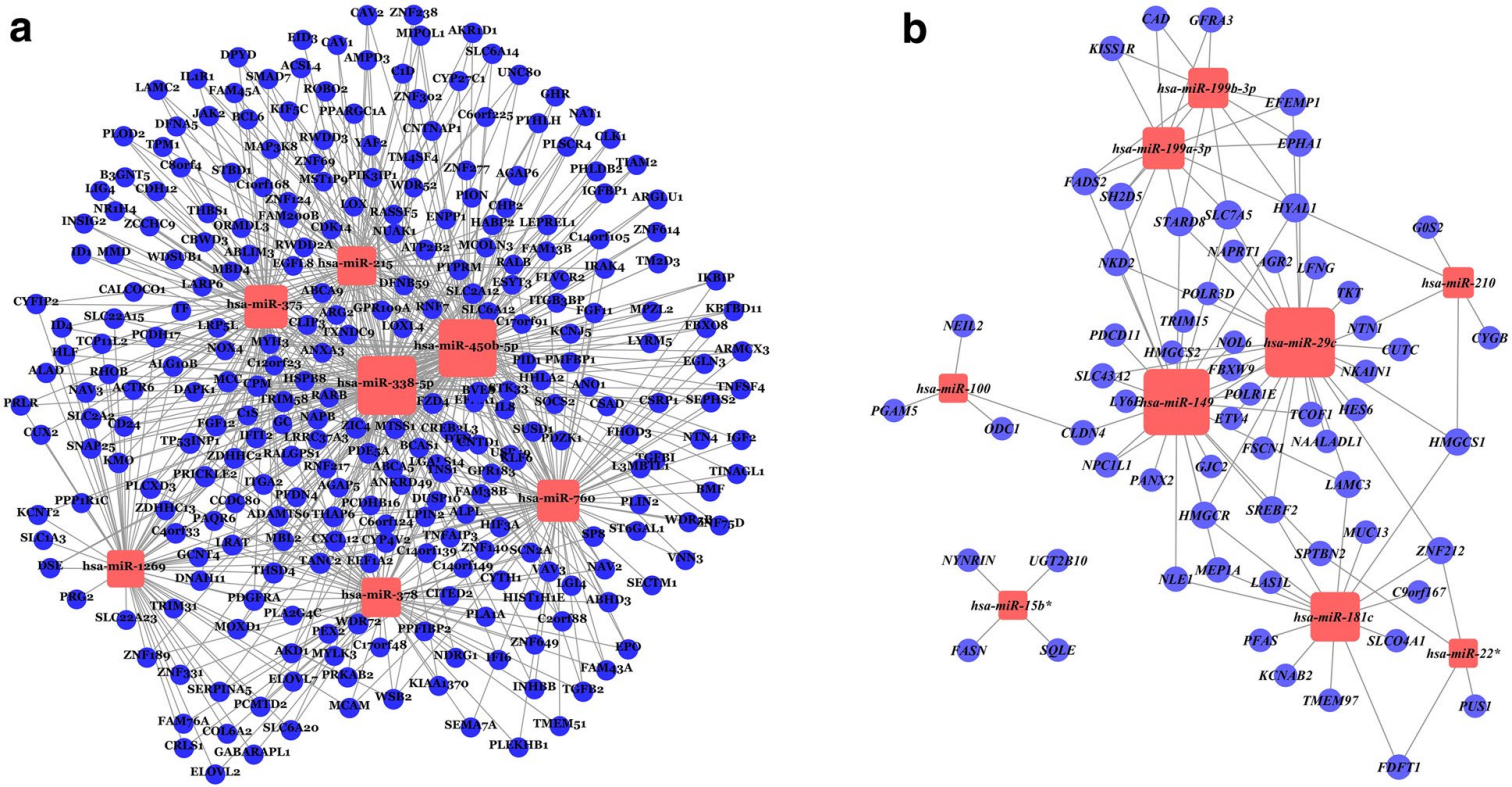
miRNA regulatory network constructed with candidate miRNA–gene interactions. **a** shows the up-regulated miRNA regulatory network, which consists of 7 up-regulated miRNAs and 274 down-regulated genes. **b** shows the down-regulated miRNA regulatory network, which consists of 9 down-regulated miRNAs and 62 up-regulated genes. *Pink* nodes represent miRNAs and *blue* nodes represent genes. The size of miRNAs represents the number of candidate target genes; edges represent the relationship between miRNAs and genes.

To investigate the function of differentially expressed miRNAs, miRNA target genes were identified and miRNA target networks were constructed. First, seven miRNA target computational prediction methods were used to predict potential miRNA target genes, while considering all human genes as potential targets. This yielded 54,933 miRNA–target interactions between 18 differentially expressed miRNAs and 15,058 genes. To verify the miRNA–gene regulatory relationship in HCSCs, differential gene expression was considered in the context of miRNA–mRNA interaction whereby 1,460 miRNA–gene interactions between 16 and 398 differentially expressed miRNAs and differentially expressed genes were selected (Table 2). Considering the mechanism of miRNA-mediated mRNA down-regulation, 651 miRNA–gene interactions between 7 up-regulated miRNAs and 274 down-regulated genes, and 103 miRNA–gene interactions between 9 down-regulated miRNAs and 62 up-regulated genes were selected (this workflow is summarized in Fig. 3). These interactions were then used as candidate miRNA–gene interactions to construct a miRNA–gene regulatory network (Fig. 4).

**Table 2:** Details of target genes regulated by differentially expressed miRNAs

miRNA	Up/down-regulation of miRNA (HCSC/HCC)	Number of target genes				
		Total predicted targets	Differentially expressed targets	Up-regulated targets (HCSC/HCC)	Down-regulated targets (HCSC/HCC)	Candidate targets
hsa-miR-100	Down	574	22	4	18	4
hsa-miR-210	Down	1180	33	5	28	5
hsa-miR-29c	Down	3533	99	26	73	26
hsa-miR-181c	Down	5492	141	15	126	15
hsa-miR-22*	Down	2343	55	4	51	4
hsa-miR-15b*	Down	1641	45	4	41	4
hsa-miR-199a-3p	Down	3707	99	11	88	11
hsa-miR-199b-3p	Down	3456	96	10	86	10
hsa-miR-149	Down	5100	116	24	92	24
hsa-miR-378d	Up	7	0	0	0	0
hsa-miR-450b-5p	Up	5865	146	8	138	138
hsa-miR-338-5p	Up	5240	156	15	141	141
hsa-miR-760	Up	4249	113	32	81	81
hsa-miR-378	Up	3486	92	20	72	72
hsa-miR-215	Up	2878	81	11	70	70
hsa-miR-375	Up	3312	95	10	85	85
hsa-miR-1269b	Up	12	0	0	0	0
hsa-miR-1269	Up	2877	71	7	64	64

Seven miRNA target computational prediction sources were used to identify the interactions of miRNAs and genes. The miRNA-target interactions which occurred in at least two of these sources were considered. For each miRNA, the expression level of the target genes in HCSC and HCC was considered. The detail result is listed in this table

The network is an objective representation of all regulatory relationships between miRNAs and genes in HCSCs. Fig. 4a, b represent the regulatory networks of miRNAs up- and down-regulated in HCSCs compared with cancer cells, respectively. From Fig. 4, it is clear that up-regulated miRNAs interact with relatively more target genes compared with down-regulated miRNAs. Hsa-miR-338-5p and hsa-miR-450b-5p, which have a large number of target genes, were the most important components in the up-regulated miRNA regulatory network. In the down-regulated miRNA regulatory network, four target genes were exclusively regulated by has-miR-15b* and the down-regulated miRNA regulatory network could be divided into two sub networks. To identify the significance of this feature, the Fisher’s exact test was used here [32]. However there was no significant difference (p = 0.1149), suggesting that this feature may be the result of random chance. The importance of this feature requires further validation.

**Fig. 5:**
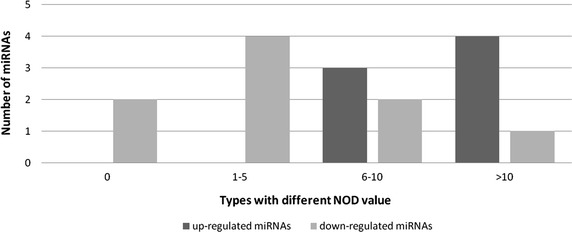
Distribution of miRNAs with different NODs. This *figure* shows the distribution of miRNAs with different NODs. NOD, defined in Zhang et al. [16], refers to the number of genes uniquely regulated by one specific miRNA. It characterizes the independent regulatory power of individual miRNAs.

Using a ‘novel out degree’ (NOD), defined by Zhang et al. [16], we measured the independent regulatory power of individual miRNAs. The NOD value represents the number of genes uniquely regulated by one specific miRNA. The distribution of miRNA NOD values is shown in Fig. 5. Consistent with the above result, there were more up-regulated miRNAs with large NOD values. The up-regulated miRNAs had larger independent regulatory power.

### Analysis of stem-cell-associated miRNA pathways

**Fig. 6:**
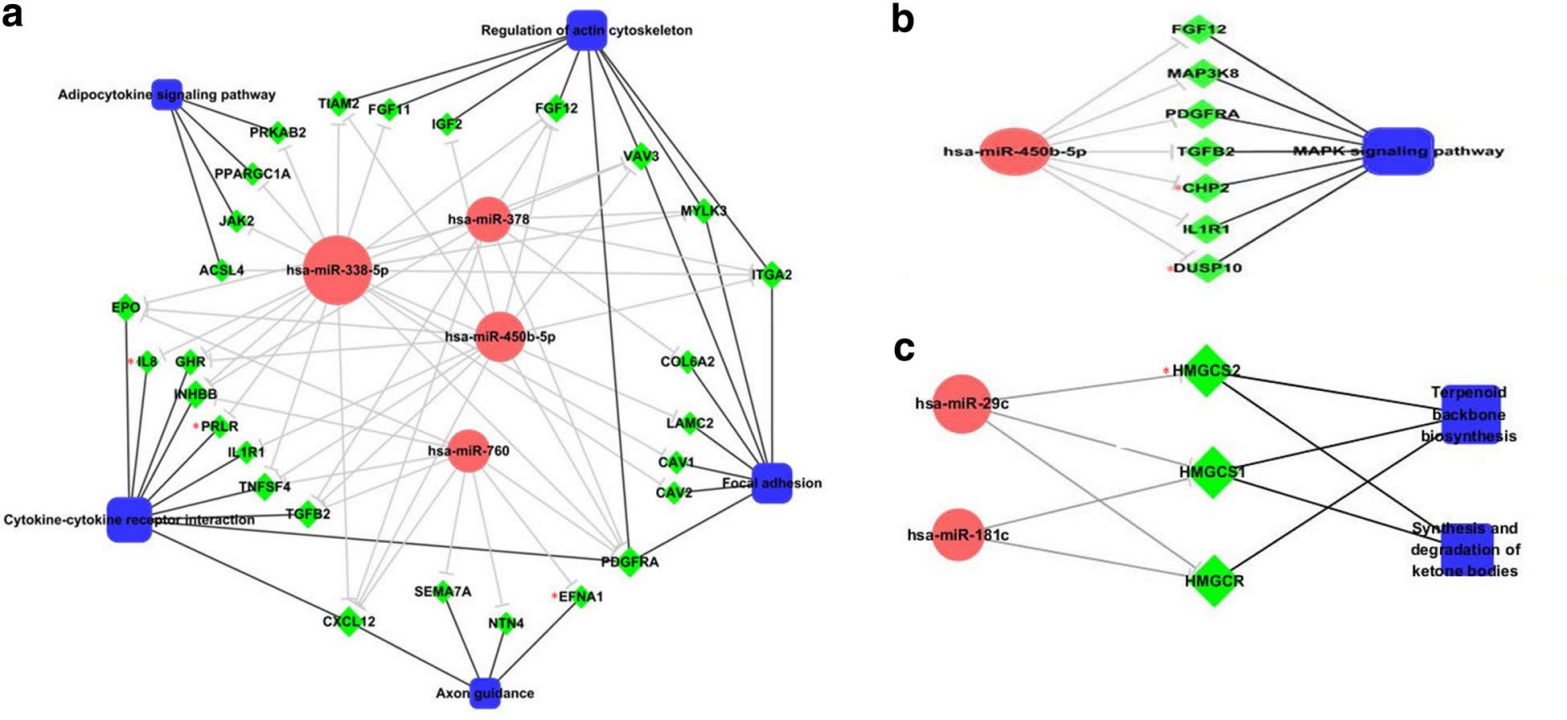
miRNA–gene–pathway regulatory networks. **a** shows the network for five pathways related to tumor invasion and metastasis. **b** shows the network for the *MAPK signalling pathway*. **c** shows the network for two new pathways, which have not been previously associated with cancer. MiRNAs, genes, and pathways are represented by nodes (*pink* miRNAs; *green* genes; and *blue* pathways). Edges of dark color represent the relationship between genes and pathways; and edges of *light color* represent the relationship between miRNAs and genes. Nodes marked with a *red* *asterisk* refer to genes uniquely regulated by one specific miRNA.

Investigating miRNA-regulated pathways should help uncover the underlying mechanisms of stem cells. Using KEGG pathways, we performed enrichment analysis to identify stem-cell-related pathways. Significantly enriched KEGG pathways (p value <0.05) are shown in Table 3. To investigate the relevance of these pathways to cancer, we searched PubMed for published papers describing the roles of these pathways; published cancer-associated-functions are listed in Table 4. Five pathways, enriched with up-regulated miRNA targets, reportedly participate in tumor invasion and metastasis; these pathways are *Cytokine*-*cytokine receptor interaction, Regulation of actin cytoskeleton, Focal adhesion, Axon guidance,* and *Adipocytokine signaling* pathway. Another important cancer-related pathway, *MAPK signaling pathway*, was also enriched with up-regulated miRNA targets. To study the function of up-regulated miRNAs in the context of these cancer-related pathways, we constructed miRNA-gene-pathway regulatory networks. Fig. 6a shows the network for the five pathways related to tumor invasion and metastasis. Fig. 6b shows the network for the *MAPK signaling pathway*, which is involved in HCC growth and survival. In these six cancer-related pathways, four genes (IL8, PRLR, EFNA1, and CHP2) were uniquely regulated by one specific miRNA each (hsa-miR-338-5p_IL8, hsa-miR-338-5p_PRLR, hsa-miR-760_EFNA1, hsa-miR-450b-5p_CHP2).

**Table 3:** Significantly enriched KEGG pathways (p-value <0.05)

miRNA	Term_ID	Term_name	Gene_count	Genes	P-value
hsa-miR-29c	hsa00900	Terpenoid backbone biosynthesis	3	HMGCR, HMGCS1, HMGCS2	0.00023
hsa-miR-29c	hsa00072	Synthesis and degradation of ketone bodies	2	HMGCS1, HMGCS2	0.014
hsa-miR-181c	hsa00900	Terpenoid backbone biosynthesis	2	HMGCR, HMGCS1	0.012
hsa-miR-338-5p	hsa05200	Pathways in cancer	10	RARB, FZD4, ITGA2, IL8, FGF12, DAPK1, EGLN3, PDGFRA, LAMC2, FGF11	0.0036
hsa-miR-338-5p	hsa04510	Focal adhesion	7	CAV1, CAV2, ITGA2, LAMC2, MYLK3, PDGFRA, VAV3	0.012
hsa-miR-338-5p	hsa04810	Regulation of actin cytoskeleton	7	VAV3, MYLK3, PDGFRA, ITGA2, FGF11, TIAM2, FGF12	0.016
hsa-miR-338-5p	hsa04920	Adipocytokine signaling pathway	4	JAK2, ACSL4, PPARGC1A, PRKAB2	0.026
hsa-miR-338-5p	hsa04060	Cytokine-cytokine receptor interaction	7	CXCL12, GHR, INHBB, IL8, PDGFRA, PRLR, TNFSF4	0.038
hsa-miR-378	hsa04510	Focal adhesion	5	COL6A2, ITGA2, MYLK3, PDGFRA, VAV3	0.0058
hsa-miR-378	hsa04810	Regulation of actin cytoskeleton	5	VAV3, MYLK3, PDGFRA, ITGA2, FGF12	0.0073
hsa-miR-378	hsa04060	Cytokine-cytokine receptor interaction	5	CXCL12, EPO, INHBB, PDGFRA, TGFB2	0.014
hsa-miR-378	hsa05200	Pathways in cancer	5	FGF12, FZD4, ITGA2, PDGFRA, TGFB2	0.03
hsa-miR-378	hsa05210	Colorectal cancer	3	FZD4, PDGFRA, TGFB2	0.038
hsa-miR-760	hsa04060	Cytokine-cytokine receptor interaction	6	CXCL12, EPO, INHBB, PDGFRA, TGFB2, TNFSF4	0.011
hsa-miR-760	hsa05200	Pathways in cancer	6	EGLN3, FGF11, FZD4, PDGFRA, TGFB2, RALB	0.027
hsa-miR-760	hsa04360	Axon guidance	4	CXCL12, EFNA1, NTN4, SEMA7A	0.03
hsa-miR-215	hsa05200	Pathways in cancer	6	FGF11, FGF12, FZD4, ITGA2, LAMC2, RALB	0.014
hsa-miR-450b-5p	hsa04060	Cytokine-cytokine receptor interaction	7	IL1R1, EPO, CXCL12, TNFSF4, TGFB2, PDGFRA, GHR	0.029
hsa-miR-450b-5p	hsa04010	MAPK signaling pathway	7	IL1R1, DUSP10, TGFB2, PDGFRA, CHP2, MAP3K8, FGF12	0.032
hsa-miR-450b-5p	hsa04810	Regulation of actin cytoskeleton	6	TIAM2, FGF12, IGF2, ITGA2, PDGFRA, VAV3	0.043

KEGG database and DAVID Bioinformatics Resources 6.7 were used for pathway enrichment analyses of genes regulated by identified HCSC miRNA biomarkers. Significant enriched pathways (P value <0.05) are listed in this table.

**Table 4:** Published cancer-associated functions of enriched pathways

Term_ID	miRNAs	Term_name	Relevance to cancer
hsa05200	hsa-miR-215,hsa-miR-338-5p, hsa-miR-378,hsa-miR-760	Pathways in cancer	
hsa04060	hsa-miR-338-5p,hsa-miR-378, hsa-miR-450b-5p,hsa-miR-760	Cytokine-cytokine receptor interaction	Cytokines can control invasion and metastasis, and also function to inhibit tumor progression [40]
hsa04810	hsa-miR-338-5p,hsa-miR-378, hsa-miR-450b-5p	Regulation of actin cytoskeleton	Several studies revealed that molecules that link migratory signals to the actin cytoskeleton are upregulated in invasive and metastatic cancer cells [41]
hsa04510	hsa-miR-338-5p, hsa-miR-378	Focal adhesion	Focal adhesion kinase, which plays an important role in tumor progression and metastasis, is overexpressed and activated in a variety of human cancers [42, 43]
hsa04010	hsa-miR-450b-5p	MAPK signaling pathway	The MAPK pathway plays an important role in HCC in that its activation is reportedly involved in HCC growth and survival [35]
hsa04360	hsa-miR-760	Axon guidance	The ligand/receptor pairs of axon guidance regulate tumor angiogenesis [44]
hsa04920	hsa-miR-338-5p	Adipocytokine signaling pathway	Adipocytokine signaling pathway has been demonstrated participate in breast cancer progression [45]
hsa05210	hsa-miR-378	Colorectal cancer	
hsa00900	hsa-miR-181c, hsa-miR-29c	Terpenoid backbone biosynthesis	
hsa00072	hsa-miR-29c	Synthesis and degradation of ketone bodies	

We searched PubMed for published papers to explain the relevance of significantly enriched pathways to cancer. The published cancer-associated-functions of these pathways are listed in this table.

However, two pathways, *Terpenoid backbone biosynthesis* (hsa00900) and *Synthesis and degradation of ketone bodies* (hsa00072), have not previously been related to cancer. These two pathways were enriched with the target genes of down-regulated miRNAs (hsa00900_hsa-miR-181c, has-miR-29c, hsa00072_hsa-miR-29c). The network of these two pathways is shown in Fig. 6c.

## Discussion

To better understand the characteristics of HCSCs established in our lab, we identified differentially expressed miRNAs and genes in HCSCs compared with hepatic cancer cells based on high-throughput sequencing datasets (9 up-regulated miRNAs; 9 down-regulated miRNAs; 115 up-regulated genes; 402 down-regulated genes). The relationship between these miRNAs and genes, and their pathway-level involvement were analyzed. With the aim of investigating regulatory mechanisms in HCSCs, we constructed regulatory networks based on candidate biomarkers and enriched pathways in HCSCs.

We found a complex relationship between differentially expressed miRNAs and genes in HCSCs. MiRNA–gene regulatory networks were constructed, and up-regulated miRNAs were found to regulate more target genes and have larger NOD values compared with down-regulated miRNAs. This implies that up-regulated miRNAs might be more important in the regulation of HCSCs. We identified two up-regulated miRNAs (hsa-miR-338-5p, and hsa-miR-450b-5p) that down-regulate hundreds of genes. The number of targets for hsa-miR-338-5p and hsa-miR-450b-5p were 141 and 138, respectively. The association of these two miRNAs with HCC and HCSCs has not previously been reported. Our results were obtained by computational methods only and further experimental validation is therefore required.

Through functional analyses we uncovered important biological processes involved in the regulation of HCSCs. Ten KEGG pathways were enriched in HCSCs. Pathway-level text mining was used to evaluate the relevance of these enriched pathways in cancer. Interestingly, five of the pathways have previously been reported to be involved in tumor invasion and metastasis. Invasion and metastasis are the main causes of cancer deaths, and are complex multi-step processes [33, 34]. Pathway analysis results are supported by the fact that the HCSCs established in our lab have much stronger invasive capability than hepatic cancer cells. Four up-regulated miRNAs (hsa-miR-338-5p, hsa-miR-450b-5p, hsa-miR-378, and hsa-miR-760) were identified to take part in HCSC invasion-related pathways. Two genes, PDGFRA and CXCL12, that were regulated by all four invasion-related miRNAs and that are involved in several invasion-related pathways, might play important roles in the regulation of this process (Fig. 6a). Another cancer-related pathway, *MAPK signaling pathway*, was also enriched with hsa-miR-450b-5p target genes. The MAPK signaling is a complex pathway involved in the regulation of a variety of growth and differentiation pathways and is reportedly involved in HCC growth and survival [35]. Four genes (IL8, PRLR, EFNA1, and CHP2) that were uniquely regulated by specific miRNAs, have been associated with HCC [36–39]. However, the regulatory mechanism of these genes in HCSCs requires further investigation. In addition to known cancer-related pathways, we also identified two novel HCSC-related pathways, *Terpenoid backbone biosynthesis* and *Synthesis and degradation of ketone bodies*. These two pathways were enriched with down-regulated miRNA target genes in HCSCs. Terpenoids are a large class of natural products consisting of isoprene units, while ketone bodies are produced by the liver from fatty acids and used peripherally as an energy source when glucose is not readily available. Their relevance to HCC and HCSCs has not been reported, and requires further validation.

We globally analyzed the molecular expression signature and regulatory mechanisms in HCSCs established in our lab. Although we have identified candidate molecular markers and important pathways in HCSCs, our results are preliminary and further experimental validation is required.

## Conclusions

Using high-throughput sequencing data sets and bioinformatics analyses, we identified miRNA and mRNA signatures of HCSCs. Additionally, we combined miRNA, mRNA, and pathway analyses to study the regulatory mechanisms in HCSCs by constructing miRNA–mRNA and miRNA–mRNA–pathway regulatory networks. The molecular markers and pathways identified herein might be used as candidate biomarkers and drug targets for the diagnosis and treatment of hepatic cancer.

## Additional files

Additional file 1:
**Table S1.** Consistently differentially expressed miRNAs. Additional file 2:
**Table S2.** Consistently differentially expressed mRNAs.

## Abbreviations

HCC: hepatocellular carcinoma
HCSCs: hepatic cancer stem cells
CSCs: cancer stem cells
KEGG: Kyoto Encyclopedia of Genes and Genomes
MAPK: mitogen-activated protein kinase
rRNA: ribosomal RNA
scRNA: small cytoplasmic RNA
snRNA: small nuclear RNA
snoRNA: small nucleolar RNA
tRNA: transfer RNA
